# Chylothorax Complicating Closure of the Ductus Arteriosus: The Second Case

**DOI:** 10.1155/2013/828014

**Published:** 2013-06-17

**Authors:** L. Ennazk, O. Echouka, R. El Houati, Y. Mouaffak, G. El Adib, D. Boumzebra, S. Younous

**Affiliations:** ^1^Department of Pediatric Intensive Care Unit, King Mohammed VI University Hospital, Marrakesh 40000, Morocco; ^2^Department of Cardiovascular Surgery, King Mohammed VI University Hospital, Marrakesh 40000, Morocco

## Abstract

Chylothorax is a possible complication of intrathoracic surgery. The diagnosis of postoperative chylothorax is easy; however, the treatment can cause problems of management because of the lack of consensus. In children, the most common causes of postoperative chylothorax are the cures for congenital heart diseases. We report the case of a two-year-old child, presenting with a chylothorax following surgery of the ductus arteriosus. Our case illustrates the treatment that must first include medical measures without delaying the surgery. The risk is the installation of nutritional and immune deficiency.

## 1. Introduction

The chylothorax is a serious complication of a thoracic surgery that requires dissection near the thoracic duct. It can be life threatening because of its respiratory, nutritional, metabolic, and immunological consequences. In children, it is usually a complication of a cardiothoracic surgery for congenital heart disease. It is easily diagnosed by simple thoracentesis. The treatment, which is not yet standardized, is based primarily on conservative measures such as pleural drainage, elimination of dietary fat, and the use of molecules designed to reduce the flow of chyle. It is only after failure of the medical strategy that the surgical treatment becomes necessary.

We report the case of a 2-year-old, hospitalized in pediatric intensive care unit (ICU) in the University Hospital of Marrakesh, for chylothorax complicating surgical closure of the ductus arteriosus and the different stages of treatment.

## 2. Case Report

**Figure 1:**
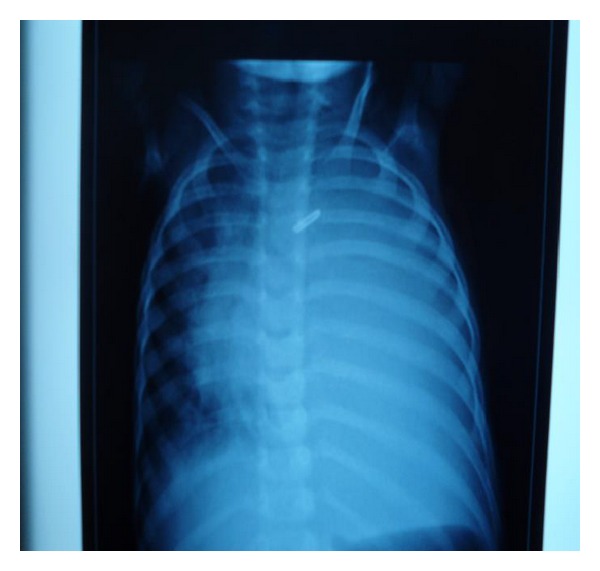
The chest X-ray showing an opaque image of pleural effusion one week after surgery.

This two years and seven months old child was operated by left thoracotomy for closure of the ductus arteriosus. She presented on the third postoperative day respiratory distress with fever. The chest X-ray showed an opaque image of pleural effusion. Pleural puncture brought back a chylous fluid. The effusion was evacuated in the surgical unit. One week later, the patient presented with signs of respiratory distress with recurrence of the chest radiographic image (Figure 1) for which she was admitted to the pediatric intensive care unit (PICU). At PICU admission, the child was conscious, pale, with a weak cry, and rapid breathing at 70 cycles per minute. His oxygen saturation was 96%. The systolic blood pressure was at 143 millimeters of mercury and the diastolic was at 85 millimeters of mercury. The child was mottled and afebrile. She weighed 7.2 kg and her height was 71 cm (-3 Standard deviation). The examination noted the pleuropulmonary left lateral thoracotomy scar, chest indrawing, and a left effusion syndrome without cardiac murmur on auscultation.

At first, symptomatic measures were implemented: half-sitting position, oxygenotherapy, infusion of saline serum before the drainage of the pleural effusion was conducted. A sample for cytological and biochemical study was made. Clinical improvement was noted after the drainage.

The biological study of the liquid confirmed the chylothorax. The level of triglyceride was 5.46 g/L (normal 0.5–2) and that of protein was at 27 g/L with a predominantly lymphocytic cytology. The bacteriological examination was negative.

Drainage has reduced 300 mL of chylous fluid and then continued to return with a speed of 50 mL/h. A total of 750 mL was reduced during the first 24 hours of drainage. Dietary measures were at the same time conducted. The patient went into diet rich in medium chain fatty acids.

During the first 48 hours, a rate of drainage of 80 mL/kg/j was noted which motivated the introduction of intravenous Octreotide by continuous infusion at a dose of 2.5 mg/kg/day. A decrease in pleural flow was noted after introduction of Octreotide up to 50 mL/kg/day then 35 mL/kg/day. The decision to start parenteral nutrition was made on the seventh day of the pleural drainage. Flows during the next ten days remained steady at around 55 mL/kg/j and 70 mL/kg/j. The decision of the revision surgery was taken on the twentieth day of the pleural drainage, on the tenth day of strict parenteral nutrition, and on the fortieth day of Sandostatin. A left thoracotomy was performed. The surgical exploration has found a breach of the thoracic duct. The act consisted of ligation of the thoracic duct. There were no signs of recurrence at follow-up visits.

## 3. Discussion

The postoperative chylothorax is defined by the presence of chyle in the pleural cavity occurring after a surgery. Etiologies of chylothorax in children are dominated by the cardiothoracic surgery [1] with a frequency estimated by Bond around 0.5 to 2% of cases. In addition, the chylothorax secondary to closure of the ductus arteriosus is exceptional. Only one case is reported by Gotsman [2] in 1966. It occurs usually after the cure of the tetralogy of Fallot, Fontan intervention, the treatment of ventricular and auricular septal defect with pulmonary stenosis, the treatment of coarctation of the aorta, and finally the ligation of the ductus arteriosus [3]. Diagnostic criteria are as follows: the milky aspect of the pleural fluid drainage, triglycerides >1.1 mmol/L, and cells >100/mL predominantly lymphocytic [4]. Although the diagnosis is easy, the treatment remains difficult. Therapeutic means are first represented by the drainage of the pleural fluid, with daily quantification of flow. A throughput less than 10 mL/Kg/j is a sign of a good evolution [5]. 

Dietary measures have a significant effect on reducing the flow in the thoracic duct chyle until its spontaneous recovery. The first is a diet low in fat but rich in medium chain fatty acids that are directly absorbed into the portal system bypassing the lymphatic drainage system [6]. In our case, for a drainage flow above 10 mL/kg/j, the decision to add Octreotide was taken. Octreotide is a somatostatin analogue which is an endogenous hormone acting on the splanchnic and the gastrointestinal tract [7]. By causing vasoconstriction of the splanchnic circulation, it causes a decrease in lymphatic flow. The time of its introduction in the management is not standardized. Surgical treatment aims to permanently stop the leakage of chyle. 

The timing of surgery remains debatable [4]; surgery should be considered when medical management of chylothorax has failed to reduce the flow of chyle and allow healing of the breach. Most authors recommend three to four weeks of medical treatment [1]. As indicated early, surgery shortens hospitalization and reduces the risk of malnutrition, and immunosuppression. There are many surgical approaches described for the ligation of the thoracic duct. If the site of the rupture is highlighted in lymphangiography, surgical ligation of the thoracic duct is the final option. The right thoracotomy is the most easy and efficient incision to ligate the thoracic duct when the patient has no situs inversus [8]. Some authors have shown the benefit of prophylactic mass ligation of the thoracic duct in patients who underwent oesophagectomy and pulmonary resection [9]. However, the interest of the prophylactic ligation has not been verified in children with congenital cardiac surgery. Recently, video-assisted thoracoscopy provides a low complication rate and an improved cost-benefit ratio [10, 11].

## 4. Conclusion

The scarcity of postoperative chylothorax in children makes the standardization of a therapeutic approach difficult. The conservative treatment remains the first therapeutic option before surgery is considered. The keeping of a register may provide a better opportunity for data analysis.
